# Anaphylactic shock during SpaceOAR Vue hydrogel procedure

**DOI:** 10.1016/j.eucr.2024.102870

**Published:** 2024-10-20

**Authors:** Lauren Nesi, Paramjot Gogia, Aishwarya Navalpakam, Nitin Vaishampayan, Conrad Maitland

**Affiliations:** a Department of Urology, Detroit Medical Center, 4160 John R St., Harper Professional Building, Suite 1021, Detroit, MI, 48201, United States; b Department of Health Sciences, Brock University, 1812 Sir Isaac Brock Way, St. Catharines, ON, L2S 3A1, Canada; c Division of Allergy, Immunology and Rheumatology, Children's Hospital of Michigan, 3901 Beaubien Blvd, Detroit, MI, 48201, United States; d Department of Oncology, Division of Radiation Oncology, Barbara Ann Karmanos Cancer Institute, 4100 John R St, Detroit, MI, 48201, United States

**Keywords:** Anaphylaxis, SpaceOAR Vue, Polyethylene glycol (PEG), Hydrogel, Prostate cancer

## Abstract

In this report, we present a unique and rare case of an intraoperative anaphylactic shock leading to cardiac arrest during the SpaceOAR Vue™ hydrogel procedure in a 70-year-old patient undergoing External Beam Radiation Therapy (EBRT) for advanced localized prostate cancer. To our knowledge, this is the first urologic case report documenting this adverse reaction associated with the placement of the SpaceOAR Vue product. We discuss the possible culprits, including the hydrogel's polyethylene glycol (PEG) and iodine content, perioperative antibiotics, and local lidocaine anesthetic, and propose relevant considerations for clinicians administering rectal hydrogel spacers.

## 1 Introduction

Allergic reactions can range from mild localized hypersensitivity responses to more severe systemic responses (i.e., anaphylaxis), and intraoperative symptoms of anaphylaxis include arterial hypotension, bronchospasm, pulselessness, oxygen desaturation, erythema, urticaria, and in more severe cases, cardiovascular collapse and cardiac arrest.**^1^** The SpaceOAR product is an absorbable, polyethylene glycol (PEG) based synthetic hydrogel spacer injected into the perirectal region to reduce the radiation dose delivered to the anterior rectum during prostate cancer (PC) radiotherapy. This hydrogel aims to protect the patient from rectal mucosal damage and has been shown to be well tolerated in PC patients while reducing rectal irradiation and toxicity.^2^^,^^3^ Based on the manufacturer's documentation, there are no specific contraindications to the placement of the SpaceOAR™ product or the newer and more recently modified SpaceOAR Vue™ Hydrogel System.^2^^,^^4^

## 2 Case presentation

### 2.1 Clinical history

A 70-year-old male recently diagnosed with stage IIIC T1cN0M0 prostate cancer (Gleason 5 + 4) elected to undergo External Beam Radiation Therapy (EBRT) and presented to our institution for transperineal placement of SpaceOAR Vue under general anesthesia. The patient has a past medical history significant for hypothyroidism, Class 1 obesity, chronic obstructive pulmonary disease (COPD), hypertension, radiculopathy, and myelopathy. His past surgical history includes multiple procedures, most notably cervical spine decompressive laminectomy in 2011 and lumbar hemilaminectomy in 2009. At the time of writing this report, the patient is on multiple medications, including amlodipine, losartan, and levothyroxine. Further, this patient has no known allergies to PEG or iodine contrast media, nor any previous allergic reactions to medications.

### 2.2 Intraoperative anaphylactic shock

The patient first received a preoperative dose of cefazolin (2 g) and was subsequently prepped and draped in a normal, sterile fashion using chlorhexidine skin preparation. The patient was hemodynamically stable during the induction of anesthesia and throughout the surgical procedure. As the case approached completion, immediately following the injection of SpaceOAR material, a significant drop in blood pressure (BP) to 56/35 mmHg was noted. Initial attempts to stabilize the patient included the administration of ephedrine (10 mg) and phenylephrine (200 mcg) intravenously, both of which failed to elicit a sufficient response. Incremental doses of epinephrine were given with minimal effect, and vasopressin (20 mg) was also administered. Due to the patient's continued instability, the laryngeal mask airway (LMA) was removed, and the patient was paralyzed and intubated. The patient received two rounds of cardiopulmonary resuscitation (CPR) due to pulseless electrical activity (PEA) before gradually regaining a pulse. A right femoral arterial line was placed under ultrasound guidance, and repeat doses of epinephrine were administered to maintain blood pressure. A transesophageal echocardiogram (TEE) was performed, revealing empty ventricles with no regional wall motion abnormalities (RWMAs). The anesthesiologist confirmed these findings. The patient was then started on an intravenous epinephrine infusion at a rate of 0.05 mcg/kg/min and return of spontaneous circulation (ROSC) was achieved. During the resuscitation efforts, the patient developed a significant urticarial rash on the torso and extremities. The rash initially appeared on the patient's trunk and rapidly spread to involve the extremities, and presented as diffuse, erythematous lesions with areas of confluence. The working diagnosis was an anaphylactic reaction, with potential triggers suspected to be the SpaceOAR suspension due to the patient's previous tolerance to anesthetic agents and preoperative antibiotics. The medical team administered steroids, diphenhydramine, and famotidine. The patient was subsequently admitted to the surgical intensive care unit (SICU) for monitoring.

### 2.3 Outcome and follow-up

Upon transfer to the SICU, the patient was extubated the same night and demonstrated a return to baseline function. Both transthoracic echocardiogram (TTE) and TEE performed at the time of arrest showed no significant cardiac events. The rash had also resolved. The patient denied any residual symptoms such as pain, nausea, vomiting, chest pain, or shortness of breath. The patient also had an acutely elevated serum tryptase level of 60.2 μg/L, which is suggestive of true anaphylaxis. The patient eventually made a full recovery and was deemed medically stable for discharge after further evaluation and monitoring, with a one-week follow-up scheduled with his primary care provider and urology. Later, the patient was evaluated outpatient by Immunology who confirmed that the patient indeed experienced anaphylaxis with an unknown trigger.

## 3 Discussion

This case report highlights a rare but significant complication associated with the use of the SpaceOAR Vue prostate-rectal hydrogel spacer product, specifically the possibility of anaphylactic reactions following the administration of the hydrogel compound. The presence of PEG in the product raises concerns, particularly for use in patients with known sensitivities or allergies to PEG.

Given that the patient had no known or documented allergy to PEG and was able to tolerate Bicalutamide in the past, which contains PEG400 as an inactive ingredient,**^5^** it may be less likely that the anaphylactic reaction was due to the PEG content within the SpaceOAR hydrogel itself. In general, most immediate anaphylactic reactions to PEG-containing compounds are induced by high-molecular-weight (HMW) PEG products and initial PEG-sensitization may be caused by low-molecular-weight (LMW) PEG pharmaceuticals (≤5 kDa).^6^, ^7^, ^8^ For context, the SpaceOAR product uses an 8-arm PEG with a molecular weight of 15 kDa (15,000 Da). This multi-arm PEG derivative is crosslinked into the hydrogel and is considered an HMW product.**^9^** LMW PEGs can be easily absorbed through cutaneous exposure and the gastrointestinal tract, and Bicalutamide's PEG content could have had sensitizing potential in this case. PEG cross-sensitization, especially to PEGylated pharmaceutical compounds, may be generally underestimated.**^7^** The patient also had no subsequent allergic symptoms with the retained hydrogel, which may be seen in patients with PEG sensitivities.

Theoretically, this allergic reaction could have been associated with the SpaceOAR product due to the hydrogel's minute iodine content (approximately 1 % by volume). It should be noted that the patient has no previously documented iodine sensitivities or allergies. However, anaphylaxis triggered by iodine within the hydrogel product would be equally unlikely, given that the iodine is covalently bonded to the PEG polymer, meaning no freeform iodine molecules are available to cause an anaphylactic reaction. It is for this reason that the SpaceOAR system is not contraindicated for placement in patients known to be allergic to iodine.**^2^**

Further, the patient tolerated the standard prophylactic antibiotic regimen administered preoperatively, to which he has historically demonstrated good tolerance in several prior surgical cases without any adverse reactions. The length of time from the administration of cefazolin until the anaphylactic shock was approximately 20 minutes. This period of time is in line with other cases of intraoperative antibiotic-induced anaphylaxis, which tend to show symptomatic onset following antibiotic administration and during the maintenance phase of anesthesia.**^10^** After neuromuscular blocking agents (NMBAs) and latex contact, antibiotics are an identifiable and leading causative agent of perioperative anaphylaxis in the surgical patient.^11^^,^^12^ Within beta-lactams, cefazolin specifically is the most frequently implicated antibiotic in cases of intraoperative anaphylaxis.**^13^** Given that cefazolin has a low cross-reactivity with penicillin and other cephalosporins due to its unique R1 side chain, it is safe for the patient to receive other cephalosporins in the future.**^14^**

Another potential cause for the anaphylactic reaction may have been the local lidocaine anesthetic. However, anaphylactic reactions to local anesthetics are overall rare, making up an incidence of less than 1 %, and IgE-mediated allergy to lidocaine is demonstrably infrequent relative to its widespread use. Similar to antibiotic administration, while the exact timing can vary, lidocaine-induced anaphylaxis can occur rapidly after administration but usually happens during the induction, preparation, or maintenance phase of anesthesia.^15^^,^^16^

It is important to note that prior tolerance to PEG, iodine, antibiotics (cefazolin), or even local anesthetics (lidocaine) does not eliminate the possibility of developing an allergy as the patient must be sensitized prior to having a reaction. Ideally, this patient would undergo full allergy testing for the suspected perioperative allergens above through skin prick and intradermal tests, potentially followed by graded challenges or dose testing under controlled conditions to definitively isolate the culprit of the anaphylactic reaction. However, the patient declined further clinical workup.

The closeness in time is more suggestive that the SpaceOAR product is the culprit. Although prior documentation of such reactions is limited, this case emphasizes the need for heightened awareness among healthcare providers on the possibility of unexpected perioperative adverse reactions involving the SpaceOAR Vue product. It is therefore critical for clinicians to continuously monitor for unexpected complications to enhance patient safety and surgical outcomes. Further research into the safety profile of PEG-containing prostate-rectal hydrogel spacers is warranted to guide clinical practice and understanding.

While there are multiple reports of adverse events related to this procedure for prostate cancer radiotherapy, including those logged in the Manufacturer and User Facility Device Experience (MAUDE) database, the most prevalent complications include acute pulmonary embolism, rectal ulcerations, and infections necessitating bowel or urinary diversions.^17^, ^18^, ^19^, ^20^ To the best of our knowledge, this is the first urologic case report documenting an anaphylactic shock as an adverse reaction to the use of the SpaceOAR Vue™ product. There is a novel opportunity to study the use and allergenic potential of this product in patients with documented PEG and/or iodine sensitivities or allergies.

## 4 Conclusion

While adverse events following the SpaceOAR hydrogel procedure are rare, severe intraoperative anaphylactic reactions are rarer still. Appropriately responding to an anaphylactic reaction during surgery is crucial yet difficult given the concurrent administration of several anesthetic agents, prophylactic antibiotic therapy, and other possible allergens. Overall, healthcare providers should regard the SpaceOAR system and hydrogel compound as a rare but possible cause of intraoperative anaphylactic shock, and further confirmatory research is warranted on allergic sensitivities to iodine, PEGylated hydrogel compounds, antibiotics, localized lidocaine anesthetics, and/or a combination of them all.

## CRediT authorship contribution statement

**Lauren Nesi:** Conceptualization, Data curation, Formal analysis, Investigation, Methodology, Project administration, Resources, Supervision, Validation, Visualization, Writing – original draft, Writing – review & editing. **Paramjot Gogia:** Conceptualization, Data curation, Formal analysis, Investigation, Methodology, Project administration, Resources, Validation, Visualization, Writing – original draft, Writing – review & editing. **Aishwarya Navalpakam:** Formal analysis, Methodology, Validation, Writing – review & editing. **Nitin Vaishampayan:** Formal analysis, Methodology, Validation, Writing – review & editing. **Conrad Maitland:** Formal analysis, Methodology, Validation, Writing – review & editing.

## Consent

We thank the patient for their participation in this case report. Consent was duly obtained from the patient for the publication of this case report.

## Data availability

Additional data regarding this case is not publicly available to protect patient anonymity.

## Disclosures & conflicts of interest

The authors have no conflict(s) of interests to disclose.

## Funding

This research did not receive any specific grant from funding agencies in the public, commercial, or not-for-profit sectors.
